# Is there any Link Between Vitamin D and Recurrence of Atrial Fibrillation after Cardioversion?

**DOI:** 10.21470/1678-9741-2019-0166

**Published:** 2020

**Authors:** Belma Yaman, Levent Cerit, Hatice Kemal Günsel, Zeynep Cerit, Songül Usalp, Ümit Yüksek, Uğur Coşkun, Hamza Duygu, Onur Akpınar

**Affiliations:** 1 Department of Cardiology, Near East Faculty of Medicine, Nicosia, Cyprus.; 2 Department of Cardiology, University of Kyrenia Faculty of Medicine, Kyrenia, Cyprus.; 3 Department of Pediatric Cardiology, Near East University, Nicosia, Cyprus.

**Keywords:** Atrial Fibrillation, Electric Countershock, Vitamin D, Prevalence, Quality of Life, Electrocardiography, Heart Atria, Hypertension

## Abstract

**Introduction:**

Atrial fibrillation (AF) is the most common chronic arrhythmia in the elderly population. In symptomatic patients, restoration and maintenance of sinus rhythm improve quality of life. Unfortunately, AF recurrence still occurs in a considerable number of patients after cardioversion (CV). In this study, we aimed to evaluate the association between vitamin D (VitD) and AF recurrence after electrical or medical CV.

**Method:**

A total of 51 patients who underwent CV for symptomatic AF were included in the study. AF recurrence was defined as an AF pattern in 12-lead electrocardiography (ECG) recording after CV within 6 months or ECG Holter recording of AF lasting more than 30 seconds at 6-month follow-up.

**Results:**

Mean vitD level was 21.4 ng/ml in our study population. VitD level was lower in the AF recurrence group than in the non-recurrence group (18 ng/ml *vs*. 26.3 ng/ml, respectively; *P*=0.001). Additionally, left atrial diameter was larger in the AF recurrence group compared to the non-recurrence group (4.4 *vs*. 4.1, *P*=0.025). Patients with AF recurrence were older than patients without AF recurrence, and, although the prevalence of hypertension is higher in the AF recurrence group, there was no statistically significant difference (*P*=0.107, *P*=0.867).

**Conclusion:**

In our study, there is a strong association between vitD level and AF recurrence after CV. VitD deficiency might be a predictor of high risk of AF recurrence after CV and vitD supplementation during the follow-up might help the maintenance of sinus rhythm.

**Table:** 

Abbreviations, acronyms & symbols				
2D	= Two-dimensional		OR	= Odds ratio
AF	= Atrial fibrillation		PAF	= Paroxysmal atrial fibrillation
BMI	= Body mass index		RAAS	= Renin-angiotensin-aldosterone system
CAD	= Coronary artery disease		SPSS	= Lactate dehydrogenase
CV	= Cardioversion		TEE	= Transesophageal echocardiography
DM	= Creatine kinase-muscle/brain		TSH	= Thyroid-stimulating hormone
ECG	= Electrocardiography		TSH	= Thyroid-stimulating hormone
ECG	= Electrocardiography		VitD	= Vitamin D
HT	= Hypertension			

## INTRODUCTION

Atrial fibrillation (AF) is the most common chronic arrhythmia in the elderly population and associated with increased risk of stroke, high morbidity and mortality^[1,2]^. Although there are several risk factors for AF, such as hypertension (HT), diabetes mellitus (DM), heart failure (HF), older age, coronary artery disease (CAD), valvular heart disease and obesity, the aetiology of AF is still largely unknown^[3,4]^. Medical and electrical cardioversion (CV) are well-established treatment modalities to restore sinus rhythm in patients with AF^[5]^. Several factors, including type of AF, patient comorbidities, structural heart disease, and anticoagulation status, should be considered deciding to perform CV^[6]^.

Restoration and maintenance of sinus rhythm improve quality of life in symptomatic patients. The procedural success of CV depends on age, AF duration, left atrial size, CAD and chronic pulmonary disease^[7,8]^. Smoking, CAD, sleep apnea syndromes and type of AF are associated with increased risk of AF recurrence^[9]^.

Vitamin D (vitD) is transformed into an active metabolite in kidney and liver and binds specific tissue receptors on intestines, bone and kidney to increase serum calcium level. VitD receptors are also found in cardiomyocytes, brain, vascular smooth muscle cells, endothelial cells, pancreas, prostate and skeletal muscles^[10]^. In recent years, vitD deficiency has been shown not only to increase the risk of skeletal system diseases, but also to have adverse effects on the cardiovascular system with regulating the renin-angiotensin-aldosterone system (RAAS) and inflammatory process. VitD regulates the expression of anti-inflammatory cytokines such as interleukin 10 (IL-10) and IL-6^[11]^. Two of the above-mentioned mechanisms are thought to be responsible for the high risk of AF in patients with vitD deficiency. In the current literature, there are inconsistent results about the association between vitD and risk of AF^[12,13]^.

In light of this knowledge, we aimed to evaluate the association between serum vitD level and risk of AF recurrence after electrical or medical CV.

## METHODS

### Study Population

Between September 2017 and October 2018, 52 retrospective patients were included in our study, admitted to our cardiology department with AF and rhythm control strategy, chosen for medical or electrical CV. Patients with thrombus in left atrial appendage or other cardiac chambers, severe mitral valve disease, obstructive sleep apnea syndrome, hyperthyroidism, acute coronary syndrome, and using any medication that could affect vitD levels were excluded from the study. Patients with permanent AF were also excluded. Transesophageal echocardiography (TEE) was performed in all patients before CV, regardless of taking anticoagulant treatment and AF duration. After a successful CV, patients were followed for six months with regard to AF recurrence. The study was approved by the local Ethics Committee, and all patients provided written informed consent.

A detailed medical history of each patient was compiled, including the history of DM, HT, hyperlipidemia, peripheral artery disease, smoking, alcohol use, family history of CAD and treatment history. The patients’ height and weight in fasting were measured; body mass indexes (BMI) (weight/height in kg/m^2^) were calculated. Fasting plasma glucose, total cholesterol, triglyceride, low-density lipoprotein cholesterol, high-density lipoprotein cholesterol, thyroid-stimulating hormone (TSH) and serum vitD levels were assayed in blood samples after 12 hours overnight fasting before CV.

### Echocardiographic Examination

The two-dimensional (2D) transthoracic echocardiography and TEE were performed to all participants before CV with available system (Vivid E9 system; General Electric Healthcare, Milwaukee, Wisconsin, USA). Standard 2D echocardiographic measurements were taken according to the guidelines of the American Society of Echocardiography and the European Society of Echocardiography^[14]^. Spontaneous echo contrast left atrial appendage thrombus and pulse wave velocities of left atrial appendage were evaluated by TEE.

### Definition of AF

Paroxysmal atrial fibrillation (PAF) is defined as an episode of AF, which ends with electrical or medical CV in less than seven days. Persistent AF is defined as an AF, which occurs longer than seven days and ends with CV^[13]^. Routine electrocardiography (ECG) was performed at each visit, and 24-hour ECG Holter monitoring was scheduled for the 6-month follow-up visit. AF recurrence is defined as an AF pattern in 12-lead ECG recording after CV within 6 months or lasting more than 30 seconds of AF attacks in ECG Holter recording at 6 months of follow-up.

### Cardioversion

Medical CV was preferred with amiodarone first with the regimen following the 300 mg intravenous (IV) bolus over 30 to 60 minutes, 1000 mg continuous IV infusion regimen up to 24 hours. The electrical CV was performed to non-responders of medical CV. Electrical CV was performed with patients in a fasting state under sedation and biphasic cardioverter-defibrillator devices between 50-200 Joules in synchronised mode. In patients with normal or low body mass index, the first shocks were done with 100 Joules, and, if the first shock is unsuccessful, the energy was increased. Also, in obese or over-weight patients, a higher energy shock was selected. To maintain sinus rhythm, amiodarone was added to treatment with 200 mg twice dayly after successful CV until hospital discharge.

### Statistical Analysis

Data were analysed using SPSS software version 17.0 (SPSS, Chicago, IL, USA). Quantitative data were presented as mean ± standard deviation. The Shapiro-Wilk test was used to test continuous variables for normality distribution. Student's t-test was used to evaluate continuous variables showing the normal distribution and Mann-Whitney U-test was used to evaluate variables that did not show normal distribution. Categorical variables were expressed as percentages. Chi-square test was used to compare categorical variables. Associations between serum vitamin D, AF recurrence, and other clinical parameters with regard to AF status were examined by univariable and multivariable logistic regression. A multivariable logistic regression model was used to derive odds ratio (OR) for the risk of AF, so that each potential confounder variable with a *P*-value ≤ 0.10 (based on univariable analysis) was entered. Statistical significance was defined as a *P*-value < 0.05.

## RESULTS

Fifty-two patients met the inclusion criteria and were included in the study; one patient was excluded due to lacking of laboratory data. Baseline characteristics of the randomised patients are listed in Table 1. There were 51 patients, with a mean age of 69.7 years, and 39.2% of them were female. Electrical CV was performed in 84.3% of the patients. After CV, the mean duration of the follow-up period was 6±2 months. In our study, AF recurrence after CV was 58.8% (n=30).

**Table 1:** Demographic features.

Clinical parameters	Mean±SD
Age (years)	69.6±7.8
Females, n (%)	20 (39.2)
HT, n (%)	26 (51)
DM, n (%)	10 (19.6)
Smoking, n (%)	5 (9.8)
Hyperlipidemia, n (%)	6 (11.8)
CAD, n (%)	22 (43.1)
PAF, n (%)	43 (84.3)
CV (electrical), n (%)	43 (84.3)
Recurrence, n (%)	30 (58.8)
VitD replacement, n (%)	6 (11.8)
Echocardiographic parameters	
LA diameter (cm)	4.36±0.5
LVH, n (%)	10 (19.6)
Laboratory parameters	
Serum creatinine (mg/dl)	0.92±0.2
TC (mg/dl)	172.4±48.5
LDL-C (mg/dl)	104.3±38.6
HDL-C (mg/d)	41.9±10.9
TG (mg/dl)	127.2±60.6
Calcium (mg/dl)	8.9±0.4
TSH (mIU/ml)	1.5±0.8
Vitamin B12 (pg/ml)	468.3±307.8
25 (OH) VitD (ng/ml)	21.4±9.0

CAD=coronary artery disease; CV=cardioversion; DM=diabetes mellitus; HDL-C=high-density lipoprotein cholesterol; HT=hypertension; LA=left atrium; LDL-C=low-density lipoprotein cholesterol; LVH=left ventricular hypertrophy; PAF=paroxysmal atrial fibrillation; TC=total cholesterol; TG=triglyceride; TSH=thyroid-stimulating hormone; VitD=vitamin D

**Fig. 1:**
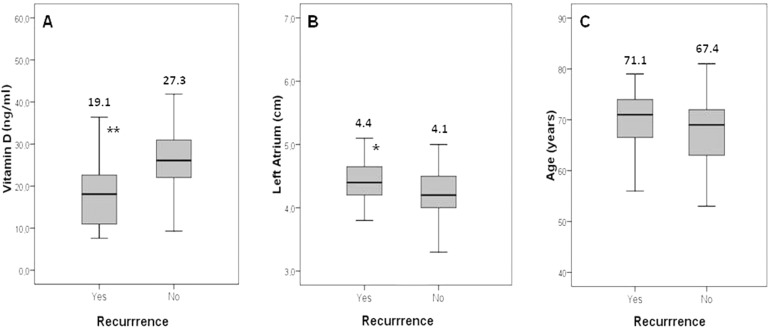
The box-plot graph shows the comparison of atrial fibrillation recurrence according to serum 25 (OH) VitD level (A), left atrium size (B), and age (C). * P<0.05, ** P<0.01.

**Fig. 2:**
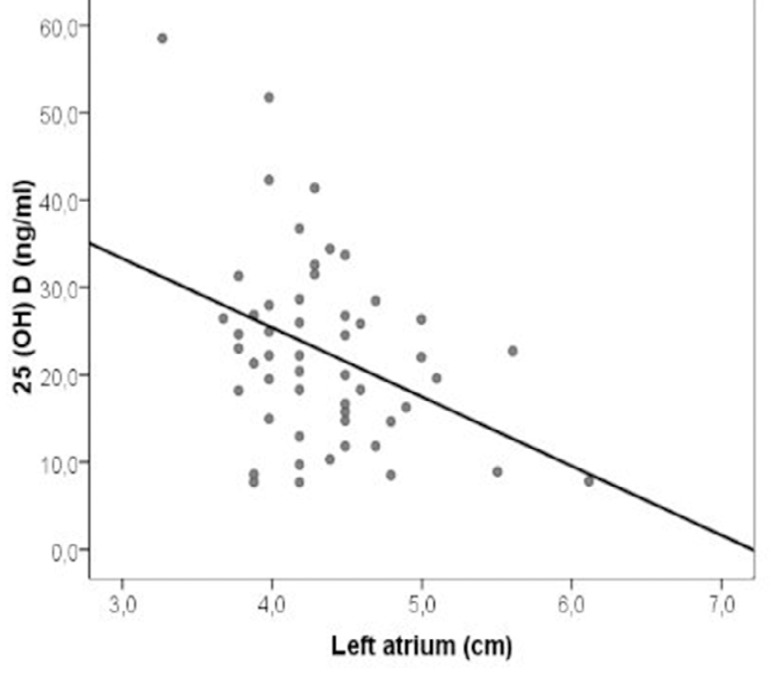
Correlation analysis shows a signficantly negative correlation between left atrial diameter and 25 (OH) VitD level.

Baseline clinical, echocardiographic and laboratory parameters were compared in AF recurrence group and non-recurrence group in Table 2. The comparison of AF recurrence according to serum vitD level, left atrial size and age were shown in Figure 1. Also, there is a negative correlation between vitD level and left atrial diameter in Figure 2 (*P*=0.006, r = -0.376).

**Table 2:** Comparison of baseline demographic features in AF recurrence or non-recurrence groups.

	Recurrence (n=30)	Non-recurrence (n=21)	*P*-value
Age (years)	71.1	67.4	0.107
Females, n (%)	13 (43.3)	7 (33.3)	0.472
HT, n (%)	15 (50)	11 (52.4)	0.867
DM, n (%)	4 (13.3)	6 (28.6)	0.177
Smoking, n (%)	3 (10)	2 (9.5)	0.955
Hyperlipidemia, n (%)	4 (13.3)	2 (9.5)	0.678
CAD, n (%)	12 (40)	10 (47.6)	0.589
PAF, n (%)	26 (86.7)	17 (81)	0.581
Electrical cardioversion, n (%)	23 (76.7)	20 (95.2)	0.073
LVH, n (%)	5 (16.7)	5 (23.8)	0.527
LA diameter (cm)	4.4	4.1	0.025
Serum creatinine (mg/dl)	0.8	0.9	0.139
TC (mg/dl)	171.9	173.2	0.929
LDL-C (mg/dl)	104.7	103.7	0.929
HDL-C (mg/dl)	40.4	44.1	0.322
TG (mg/dl)	128	126.9	0.950
Calcium (mg/dl)	8.8	9.1	0.114
TSH (mIU/ml)	1.6	1.3	0.328
Vitamin B12 (pg/ml)	456.9	485.5	0.764
25 (OH) VitD (ng/ml)	18	26.3	0.001

CAD=coronary artery disease; DM=diabetes mellitus; HDL-C=high-density lipoprotein cholesterol; HT=hypertension; LA=left atrium; LDL-C=low-density lipoprotein cholesterol; LVH=left ventricular hypertrophy; PAF=paroxysmal atrial fibrillation; TC=total cholesterol; TG=triglyceride; TSH=thyroid-stimulating hormone; VitD=vitamin D.

Left atrial diameter was larger in AF recurrence group when compared to the non-recurrence group (4.4 cm *vs*. 4.1 cm, *P*=0.025). Patients with AF recurrence were older, and HT was frequently seen in the AF recurrence group; however, there was no statistically significant difference (*P*=0.107, *P*=0.867, respectively). The prevalence of PAF was 84.3% (n=43), persistent AF was 15.7% (n=8) in our study, and AF recurrence was similar between the two groups (*P*=0.581). In addition, when comparing CV modalities, the prevalence of AF recurrence was higher in electrical CV group than in medical CV group; however, there was no statistically significant difference (*P*=0.073). Comparison of baseline demographic features in electrical CV and medical CV groups was shown in Table 3.

**Table 3:** Comparison of baseline demographic features in the electrical CV and medical CV groups.

	Electrical CV (n=43)	Medical CV (n=8)	*P*-value
Age (years)	69.3	71.2	0.635
Female gender, n (%)	14 (32.6)	6 (75)	0.024
HT, n (%)	23 (53.5)	3 (37.5)	0.406
DM, n (%)	8 (18.6)	2 (25)	0.676
Smoking, n (%)	4 (9.3)	1 (12.5)	0.780
Hyperlipidemia, n (%)	5 (11.6)	1 (12.5)	0.944
CAD, n (%)	21 (48.8)	1 (12.5)	0.057
Paroxysmal AF, n (%)	36 (83.7)	7 (87.5)	0.787
LVH, n (%)	8 (18.6)	2 (25)	0.676
LA diameter (cm)	4.3	4.4	0.606
Serum creatinine (mg/dl)	0.9	0.7	0.058
TC (mg/dl)	170.1	182.6	0.414
LDL-C (mg/dl)	104	105.6	0.726
HDL-C (mg/dl)	40.3	48.7	0.498
TG (mg/dl)	124.4	141.1	0.515
Calcium (mg/dl)	9.0	8.9	0.135
TSH (mIU/ml)	1.4	1.9	0.934
Vitamin B12 (pg/ml)	496.1	339.7	0.095
25 (OH) VitD (ng/ml)	21.8	19.4	0.103
AF recurrence n (%)	23 (53.5)	7 (87.5)	0.073

AF=atrial fibrillation; CAD=coronary artery disease; CV=cardioversion; DM=diabetes mellitus; HDL-C=high density lipoprotein cholesterol; HT=hypertension; LA=left atrium; LDL-C=low density lipoprotein cholesterol; LVH=left ventricular hypertrophy; TC=total cholesterol; TG=triglyceride; TSH=thyroid-stimulating hormone; VitD=vitamin D

**Fig. 3:**
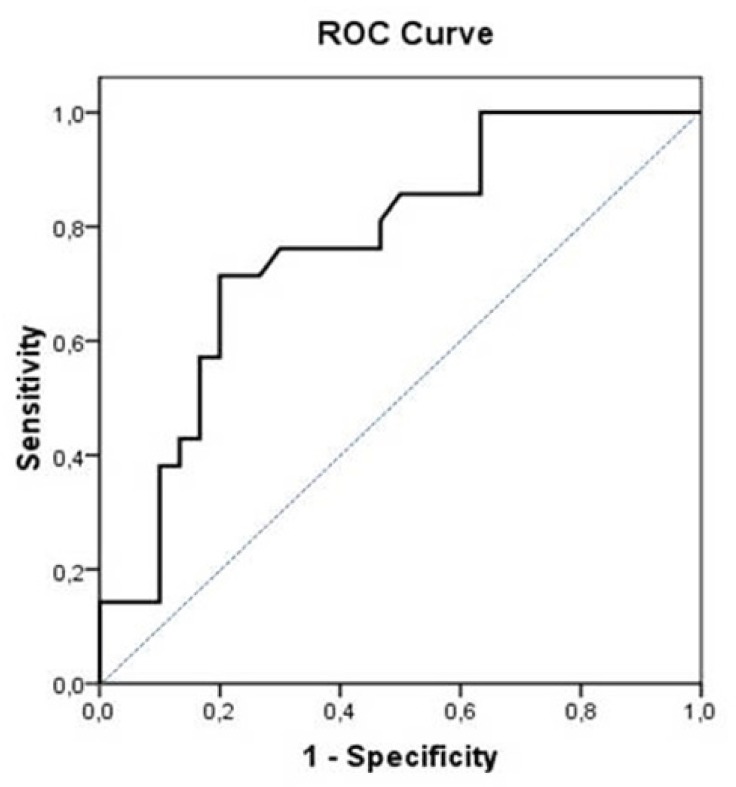
The receiver operating characteristic curve represents the cut-off point of 25 (OH) VitD level in predicting AF recurrence after CV.

The mean vitD level was 21.4 ng/ml in our study population. The vitD level was lower in the AF recurrence group than the non-recurrence group (18 ng/ml *vs*. 26.3 ng/ml, *P*=0.001). Receiver operating characteristic curve was used to explore the relationship between vitD level, which was measured before CV and AF recurrence after CV. The area under the curve was 0.745. Using a cut-off level of <22.0 ng/ml, the pre-CV vitD level was associated with prediction of AF recurrence after CV with a sensitivity of 76.2% and specificity of 67.7% (Figure 3). Univariate and multivariate analysis of basal demographic features showed a correlation between vitD and AF recurrence after CV (Table 4).

**Table 4:** Univariate and multivariate analysis of baseline demographic features.

	Univariate			Multivariate		
	B	SE	*P*	B	SE	*P*
Age (years)	–0.70	0.48	0.149			
LA diameter (cm)	–1.213	0.873	0.165			
Serum creatinine (mg/dl)	3.117	2.026	0.124			
25 (OH) VitD (ng/ml)	0.100	0.046	0.031	0.121	0.042	0.004

LA=left atrium; VitD=vitamin D

## DISCUSSION

In our study, lower serum vitD levels were found in the AF recurrence group, and vitD is an independent predictor of AF recurrence after CV. Another finding of our study is that the left atrial size was larger in the AF recurrence group.

VitD deficiency is known to play a key role in the pathogenesis of CAD, left ventricular hypertrophy and AF^[11,15,16]^. The vitD deficiency has been shown to be associated with left atrial fibrosis and increased risk of AF recurrence after ablation^[17]^. But this is the first study in the literature to evaluate the role of vitD deficiency in AF recurrence after CV.

The exact mechanisms of vitD deficiency in the cardiovascular system are not well-established. VitD has anti-oxidant and anti-inflammatory effects with regulating anti-inflammatory cytokines, including IL-10 and IL-6^[18,19]^. Besides that regulating calcium metabolism, vitD has electromechanical effects in the left atrium^[20]^. The link between vitD deficiency and development of AF, CAD and HT is explained with the decrease of anti-inflammatory effects in cardiac myocytes. Structural, electrical and contractile functions play a role in AF development. Left atrial fibrosis is the main mechanism of structural remodelling which progresses to AF. The association between left atrial fibrosis and vitD was found in a previous study by Canpolat et al.^[17]^. Oxidative stress, inflammation, and RAAS activation are the main mechanisms responsible for atrial fibrosis and might be associated with AF recurrence after ablation in patients with vitD deficiency^[21]^. Additionally, preoperative vitD supplementation could prevent postoperative AF after coronary artery bypass surgery^[22]^.

CV is the main treatment modality in symptomatic AF patients. AF ablation is another treatment modality to maintain sinus rhythm. After CV and ablation, a large part of patients fails to maintain sinus rhythm at follow-up^[23]^. AF type is the most important factor in predicting AF recurrence, such as permanent AF, with a high risk of AF recurrence after ablation; however, patient characteristics such as age, sex, HT, smoking, and CAD were not associated with AF recurrence after AF ablation^[9]^. In our study, although HT and advanced age are associated with AF recurrence after CV, there was no statistically significant difference. Besides that, obstructive sleep disorder may cause AF recurrence after CV or ablation with several mechanisms, such as nocturnal hypoxemia and sympathetic nervous system activity, which can activate atrial stretching and diastolic dysfunction^[24]^. Patients with obstructive sleep apnea syndrome were excluded from our study.

Left atrial size was also shown to be an important factor in predicting AF recurrence. Patients with left atrial size greater than 4 cm have a higher AF recurrence risk^[25]^. In our study, the mean left atrial diameter was 4.4 cm in the recurrence group, which was significantly larger than in the non-recurrence group.

### Limitations

Our study results should be evaluated as a part of several limitations; first, patients were taking different types of antiarrhythmic drugs; there was no homogenization in drug classes, which may affect the AF recurrence. Second, the duration between the first diagnosis of AF to CV is unknown in the paroxysmal AF group. Third, we did not take a blood sample for vitD and perform control echocardiography at six months of follow-up. The small sample size is another limitation of the study.

## CONCLUSION

Lower vitD levels are associated with increased risk of AF recurrence after CV. Due to the close association between vitD deficiency and increased risk of AF recurrence, considering vitD levels before CV might be beneficial for the maintenance of sinus rhythm, and the vitD replacement in patients with vitD deficiency may prevent AF recurrence after CV.

**Table:** 

Author's roles & responsibilities	
BY	Substantial contributions to the conception or design of the work; or the acquisition, analysis, or interpretation of data for the work; drafting the work or revising it critically for important intellectual content; final approval of the version to be published
LC	Substantial contributions to the conception or design of the work; drafting the work or revising it critically for important intellectual content; final approval of the version to be published
HK	Substantial contributions to the conception or design of the work; or the acquisition, analysis, or interpretation of data for the work; drafting the work or revising it critically for important intellectual content; final approval of the version to be published
ZC	The acquisition, analysis, or interpretation of data for the work; drafting the work or revising it critically for important intellectual content; final approval of the version to be published
SU	The acquisition, analysis, or interpretation of data for the work; drafting the work or revising it critically for important intellectual content; final approval of the version to be published
UY	Substantial contributions to the conception or design of the work; or the acquisition, analysis, or interpretation of data for the work; drafting the work or revising it critically for important intellectual content; final approval of the version to be published
UC	Substantial contributions to the conception or design of the work; drafting the work or revising it critically for important intellectual content; final approval of the version to be published
HD	Substantial contributions to the conception or design of the work; drafting the work or revising it critically for important intellectual content; final approval of the version to be published
OA	Substantial contributions to the conception or design of the work; drafting the work or revising it critically for important intellectual content; final approval of the version to be published
